# Association of homocysteinaemia with hyperglycaemia, dyslipidaemia, hypertension and obesity

**DOI:** 10.5830/CVJA-2013-059

**Published:** 2013-10

**Authors:** Mpho Moraba, Dudu Sengwayo, Shirley Motaung

**Affiliations:** Department of Medical Science, Health Public and Health Promotion, School of Health Sciences, Faculty of Health Sciences, University of Limpopo (Turfloop Campus), Sovenga, South Africa; Department of Biomedical Sciences, Faculty of Science, Tshwane University of Technology, Pretoria, South Africa; Department of Biomedical Sciences, Faculty of Science, Tshwane University of Technology, Pretoria, South Africa

**Keywords:** hyperglycaemia, hypertriglyceridaemia, hypercholesterolaemia, hypertension, obesity, homocysteine

## Abstract

**Aim:**

Hyperhomocysteinaemia and the metabolic syndrome are associated with increased cardiovascular risk. We investigated whether there is a link between the metabolic syndrome or its components and homocysteine levels in a population without cardiovascular disease.

**Methods:**

From the population sample of 382 participants (286 females and 96 males) we isolated those reflecting the metabolic syndrome and determined their homocysteine levels. We then evaluated the association of homocysteine with hyperglycaemia, hypertriglyceridaemia, hypercholesterolaemia, hypertension and obesity, using a significance level of *p* = 0.05. Enzymatic methods were used for all biochemical parameters.

**Results:**

We found the statistical relationship between homocysteine and the metabolic syndrome as follows: hyperglycaemia (*p* = 0.175), hypertriglyceridaemia (*p* = 0.442), hypercholesterolaemia (*p* = 0.480), obesity (*p* = 0.080); and hypertension: systolic pressure (*p* = 0.002) and diastolic pressure (*p* = 0.033).

**Conclusion:**

We found no statistically significant association between baseline plasma homocysteine levels and the metabolic syndrome, except for hypertension.

## Abstract

Diabetes mellitus is a group of metabolic diseases characterised by hyperglycaemia, resulting from defects in insulin secretion, insulin action or both. It is associated with several cardiovascular disorders, including angiopathy and platelet hyperactivity, which are major causes of morbidity and mortality in type 2 diabetes mellitus.^1^ Atherosclerosis is substantially more prevalent and progresses rapidly in diabetes mellitus.^2^

There are an estimated 23.6 million people in the USA (7.8% of the population) with diabetes.^1^ The vascular complication of diabetes mellitus, at its earliest stage, is manifested as endothelial dysfunction,^3^ decreasing the bioavailability of nitric oxide, which protects blood vessels from endogenous injuries.^4^ Hyperglycaemia inhibits fibrinolysis by decreasing the activity of plasminogen activator and enhances coagulation by activating procoagulants into thrombosis.^5^

Homocysteine is an amino acid derived from methionine. The latter is an intermediate in the conversion of homocysteine to cysteine. Homocysteine is metabolised via two pathways: remethylation, in which homocysteine is converted into methionine, and transulphuration, in which homocysteine is converted into cysteine. In the former pathway, homocysteine acquires a methyl group, either from the conversion of 5-methyltetrahydrofolate into hydrofolate or from the conversion of betaine into the N′ N-dimethylglycine.^6^ Vitamins B_12_ and B_6_ are important in the conversion of 5-methyltetrahydrofolate into hydrofolate and therefore for the remethylation pathway and the metabolism of homocysteine into methionine.^7^

Epidemiological studies suggest hyperhomocysteinaemia to be an independent risk factor for developing atherothrombotic vascular disease.^8^ Mechanisms by which hyperhomocysteinaemia causes vascular disease include promotion of atherosclerosis by damaging the inner lining of arteries and promoting thrombosis through pathological collagen activation of the intrinsic pathway,^9^ impairment of thrombolysis, increased production of hydrogen peroxide, endothelial dysfunction, and increased oxidation of low-density lipoproteins.^8^

Some of the complications of arterial thrombosis following hyperhomocysteinaemia include coronary heart disease, myocardial infarction, stroke, peripheral vascular disease, miscarriage, pulmonary embolism, retinal embolism and neural tube defect (spina bifida).^9^ The homocysteine level may be increased in hypertensive, overweight and obese subjects.^10^

Homocysteine is thought to help regulate glucose metabolism and insulin absorption.^11^ Homocysteine has been suggested to contribute to the atherosclerotic process of diabetes mellitus. High homocysteine levels have been reported in diabetic patients,^2^,^12^ and elevated levels are a strong risk factor in these patients.^1^ The elevation occurs particularly in patients with type 2 diabetes, as well as in individuals in prediabetic states who exhibit insulin resistance.^13^ The levels of homocysteine in such individuals are also influenced by their insulin concentrations, and therapy with insulin and medications such as metformin and glitazones that can either raise or lower homocysteine levels.^12^

The effect of hyperhomocysteinaemia on diabetes and insulin resistance has been reported with unclear synergism.^12^ Homocysteine levels have been reported as either low or elevated compared to non-diabetic subjects, reflecting the potential role of homocysteine in the development of macro- and microvascular disease in diabetic patients.^13^,^1^ Shaikh *et al*. found that 58% of their diabetic participants had elevated homocysteine levels and males were predominant in this group.^1^ This finding is consistent with that of Schalinske’s study.^14^

These authors reported a strong association between atherosclerosis, hyperhomocysteinaemia and type 2 diabetes in the Japanese population. They concluded that hyperhomocysteinaemia in diabetes mellitus may contribute to the development of chronic complications. Vayá *et al*. established a borderline statistically significant association (*p* = 0.008) between hyperhomocysteinaemia and hyperglycaemia (*p* = 0.054).^15^

Hypertension is a condition where the artery walls are stiffer and present increased resistance to blood flow. This requires the heart to beat more forcefully and increases the pressure of blood leaving the heart. High blood pressure is often called the silent killer because in the initial stages it presents with no symptoms. It is only after an organ in the body has been irritated or damaged, that the consequences of high blood pressure are realised.^16^

Hypertension places stress on the target organs, including kidneys, eyes and heart, causing them to deteriorate over time. Hypertension contributes to 75% of all strokes and heart attacks.^17^ One in three African-Americans has hypertension. One African-American dies every hour from the disease, and more than 30% of African-Americans can count hypertension or its complications as the leading cause of death.^17^

The hypothesis that homocysteine may play a role in the pathogenesis of essential hypertension is based on the fact that homocysteine induces arteriolar constriction, renal dysfunction and increased sodium reabsorption, increasing arterial stiffness.^18^ Homocysteine increases oxidative stress, which causes oxidative injury to the vascular endothelium, diminishing vasodilation by nitric oxide, stimulating proliferation of vascular smooth muscle cells and altering the elastic properties of the vascular wall, leading to an increase in hypertension.^18^ These authors concluded that homocysteine may contribute to blood pressure elevation. Atif *et al.* observed that plasma homocysteine was raised in most patients with hypertension.^19^ The authors found in their study that 80% of their hypertensive subjects were hyperhomocysteinaemic.

Karatela and Sainani found a high prevalence of hyperhomocysteinaemia associated with raised blood pressure, with raised systolic and diastolic pressures.^10^ Nabipour *et al.* reported significantly higher homocysteine levels in subjects with high blood pressure.^20^ Vayá *et al.* however found no statistically significant association (*p* = 0.008) between hyperhomocysteinaemia and hypertension (*p* = 0.229).^15^

In large community-based studies, plasma homocysteine was found to be cross-sectionally associated with blood pressure, especially systolic pressure, unadjusted for gender and age.^21^,^22^ The authors however found that adjusted for gender and age, the relationship of plasma homocysteine to the incidence of hypertension was statistically non-significant.

Experimental investigations evaluating the association of homocysteine and blood pressure have not yielded consistent results. Diet-induced hyperhomocysteinaemia has been demonstrated to elevate blood pressure in some investigations but to lower it in others.^21^ A positive association of total homocysteine with both systolic and diastolic blood pressure was reported in several clinical cross-sectional studies.^21^ These authors found no major relationship between baseline plasma homocysteine level and incidence of hypertension.

Lipids are a group of organic compounds that include, among others, cholesterol, triglycerides, phospholipids, lipoprotein and sterols, which are insoluble in water but soluble in non-polar organic solvents.^23^ Fats (solid lipids) constitute approximately 34% of the energy used in the human body.^24^-^26^ Of the lipids, triglycerides and cholesterols [very low-density lipoprotein (LDL), LDL and high-density lipoprotein (HDL) cholesterol] are the components that play a major role in atherosclerosis, the forerunner of arteriosclerosis.^27^

All body cells are capable of LDL cholesterol (LDL-C) synthesis. This favours deposition of cholesterol in the cells and blood vessels. LDL-C is therefore atherogenic. HDL transports cholesterol from the cells to the liver for degradation into bile salts (sodium taurocholate and deoxycholate).^23^ HDL-C is therefore anti-atherogenic and protective against the development of atherothrombosis.

High triglyceride levels are significant risk factors for cardiovascular disease and are a marker for atherogenic remnant lipoprotein, such as very LDL-C. Even in the presence of tightly controlled LDL-C levels, evidence indicates that high triglyceride levels and low HDL-C levels are independent thrombosis and cardiovascular risk factors.^28^ About half of all deaths in developed countries are caused by homocysteinaemia and dyslipidaemia (hypercholesterolaemia and hypertriglyceridaemia).^29^

According to Rima and Wolfgang, there is an association between hyperhomocysteinaemia and dyslipidaemia, and diabetes mellitus is common to hyperhomocysteinaemia and hypercholesterolaemia.^30^ Vayá *et al.* found no statistically significant association (*p* = 0.008) between hyperhomocysteinaemia and low HDL-C levels (*p* = 0.491) and hypertriglyceridaemia (*p* = 0.490).^15^ However, Nabipour *et al.* found subjects with lower HDL-C levels had higher homocysteine levels (*p* = 0.001).^20^

Obesity is characterised by excess body fat due to an imbalance between calorie intake and expenditure. Causes of obesity include high calorie intake, lack of exercise and genetic susceptibility or psychiatric illness.^31^ Obesity is defined as a body mass index (BMI) greater than 30 kg/m^2^.^32^

Two patterns of obesity are central (visceral) obesity and peripheral obesity. The former is more common in males and carries a higher risk of coronary heart disease, as well as various forms of metabolic derangement, including dyslipidaemia and impaired glucose tolerance. Peripheral obesity is when fat accumulates in the gluteo-femoral area. It is more common in women but less associated with cardiovascular risk, as a complication of arterial thrombosis.^33^ Obesity is an independent risk factor for the complications of atherosclerotic vascular disease, such as myocardial infarction and stroke and has been found to elicit and increase the risk of arterial thrombosis.^6^,^34^

Obesity affects about 1.3 billion people worldwide, and 3.0 to 20.4% of South African males and 25.9 to 54.3% of females.^32^,^35^ Karatela and Sainani observed an increased prevalence of hyperhomocysteinaemia in overweight and obese subjects.^10^ Nabipour *et al.* found no significant association between homocysteine level and BMI in a study of the relationship between the metabolic syndrome and homocysteine levels.^20^ However, Vayá *et al.* found in four studies that increased homocysteine levels were related mostly to abdominal obesity.^15^

Sanlier and Yabanci found increased body weight to be associated with hyperhomocysteinaemia, but without gender differences.^36^ El-Sammak *et al.* also found hyperhomocysteinaemia to increase with age, possibly because of the presence of other factors that raise plasma total homocysteine levels with age, especially increased deterioration in other organ functions.^37^

## Methods

The study was cross-sectional and prospective. Participants were recruited by trained field workers and consented voluntarily in writing. Ethical approval was obtained from the Tshwane University of Technology Ethics Committee (Ref: 2010/09/004). A standard informed consent form was signed by all participants.

A questionnaire was used to obtain information on demographic characteristics, lifestyle, eating habits, health conditions such as surgical operations, diabetes mellitus, previous arterial thrombosis, previous pulmonary embolism, hyperlipidaemia, kidney problems, obesity/overweight and heart failure. Cardiovascular disease was one of the ailments that no participants reported to be suffering from.

Fasting blood samples were collected from participants at the Nobody Clinic in Ga-Mothapo. Subjects who had not fasted for at least nine hours before sample collection and could not withdraw medication for that period were excluded from the study.

Blood was collected by professional nurses. One 4.5-ml blood sample was collected from each participant in a sodium fluoride tube for glucose analysis, in a plain tube for triglycerides and cholesterol estimation, and in an EDTA-anticoagulated tube for homocysteine level assay.

The body weight of the participants wearing light clothing without shoes was measured using a weight scale from Omron. The height was measured without shoes in an upright position using the Seca telescopic height-measuring rod. The BMI was calculated using the formula: BMI = weight in kg/(height in m)^2^.

Blood pressure was measured using the Omron MI-5. Blood glucose, triglyceride and cholesterol levels were measured using the ILab 300 Plus Chemistry System from Beckman Coulter. Homocysteine was estimated using the Beckman Coulter Synchron system analyser. Enzymatic methods were used for all biochemical parameters.

The diagnostic criteria used for the parameters were set as follows: hyperhomocysteinaemia = blood homocysteine > 15 μmol/l, hyperglycaemia = blood glucose > 7.0 mmol/l, hypercholesterolaemia = blood cholesterol > 5.7 mmo/l, hypertriglyceridaemia = blood triglyceride > 2.26 mmol/l, obesity = BMI > 30 kg/m^2^, systolic blood pressure > 140 mmHg = hypersystolic blood pressure, and diastolic blood pressure > 90 mmHg = hyperdiastolic.

The collected data were analysed with Statistical Package for Social Science (SPSS) version 18. The results were expressed in percentages of *p*-values for association. A *p*-value of 0.05 was regarded as statistically significant.

## Results

The study consisted of 382 participants. The mean age of the study participants was 38.45 years. The mean values for the studied parameters were as follows: homocysteine 9.44 μmol/l, glucose 5.42 mmol/l, systolic blood pressure 125.65 mmHg, diastolic blood pressure 81.06 mmHg, cholesterol 4.18 mmol/l, triglycerides 1.22 mmol/l and BMI 26.80 kg/m^2^ (Table 1).

**Table 1:** Characteristics Of The Participants

*Variable*	*Mean ± SD*
Age (years)	38.45 ± 17.283
Homocysteine (µmol/l)	9.44 ± 4.13
Glucose (mmol/l)	5.42 ± 2.555
Systolic blood pressure (mmHg)	125.65 ± 19.164
Diastolic blood pressure (mmHg)	81.06 ± 11.351
Cholesterol (mmol/l)	4.18 ± 1.396
Triglycerides (mmol/l)	1.22 (0.83–1.68)
Body mass index (kg/m^2^)	26.80 ± 6.20

The associations of hyperhomocysteinaemia with hyperglycaemia (*p* = 0.175), hypertriglyceridaemia (*p* = 0.442) and hypercholesterolaemia (*p* = 0.480) were statistically insignificant. The association of hyperhomocysteinaemia with obesity was found to be partially significant (*p* = 0.080). The associations of hyperhomocysteinaemia with hypersystolic (*p* = 0.002) and hyperdiastolic (*p* = 0.033) blood pressures were statistically significant.

Of the 45 hyperglycaemic participants, three were also hyperhomocysteinaemic, constituting about 6.7%. Of the 39 hypertriglyceridaemic participants, three were also hyperhomocysteinaemic, constituting about 7.7%. Of the 38 hypercholesterolaemic participants, five were also hyperhomocysteinaemic, constituting about 13.1%. Of the 72 participants with high systolic blood pressure, 11 were also hyperhomocysteinaemic, constituting about 15.3%. Of the 84 participants with high diastolic blood pressure, 16 were also hyperhomocysteinaemic, constituting about 19.0%. Of the 95 obese participants, 10 were also hyperhomocysteinaemic, constituting about 10.5%.

## Discussion

We estimated homocysteine levels in 45 hyperglycaemic subjects for evaluation of association and found no statistical significance (*p* = 0.175) (Table 2). Three hyperglycaemic subjects (6.7%) were hyperhomocysteinaemic (Table 3). Different findings about the relationship have been reported above.

**Table 2:** *P*-Values For Significance Of Association

*Homocysteinaemia*	*Metabolic disorder*	p*-value*
*n* = 45	Hyperglycaemia (*n* = 45)	0.175
*n* = 39	Hypertriglyceridaemia (*n* = 39)	0.442
*n* = 38	Hypercholesterolaemia (*n* = 38)	0.480
*n* = 72	Systolic blood pressure (*n* = 72)	0.002
*n* = 84	Diastolic blood pressure (*n* = 84)	0.033
*n* = 95	Obesity (*n* = 95)	0.080

95% confidence interval and *p* = 0.05 level of significance.

**Table 3:** Prevalence Of Hyperhomocysteinaemia With Hyperglycaemia, Hypertriglyceridaemia, Hypercholesterolaemia, Hypertension And Obesity

*Hyperhomocysteinaemia*	*Metabolic disorder*	*Prevalence rate (%)*
*n* = 3	Hyperglycaemia (*n* = 45)	6.7
*n* = 3	Hypertriglyceridaemia (*n* = 39)	7.7
*n* = 5	Hypercholesterolaemia (*n* = 38)	13.1
*n* = 11	Systolic blood pressure (*n* = 72)	15.3
*n* = 16	Diastolic blood pressure (*n* = 84)	19.0
*n* = 10	Obesity (*n* = 95)	10.5

Prevalence of hyperhomocysteinaemia = number of hyperhomocysteinaemic subjects per number of subjects in the respective components of the metabolic syndrome.

Vayá *et al.*, in their study of the relationship between homocysteine and hyperglycaemia, found a partial association.^15^ Elias and Eng, and Shaikh *et al.* reported that homocysteine levels can be low or elevated in diabetes mellitus.^1^,^13^ These findings and ours are contrary to the findings of Mishra *et al.*^2^ and Akali *et al.*^12^ who found high homocysteine levels in diabetic patients. They found high levels of homocysteine to be a strong risk factor in diabetic patients. This was supported by the findings of Shaikh *et al.* and Schalinske.^1^,^14^

Shaikh *et al.* found more than half of their diabetic participants had elevated homocysteine levels.^1^ The discrepancy with our results could have been attributable to the influence on homocysteine of insulin concentrations, therapy with insulin and medication.^12^ Control of these confounding factors in our study may have improved the level of association.

We determined homocysteine levels in 39 hypertriglyceridaemic and 38 hypercholesterolaemic subjects. No statistically significant association was found between homocysteine and hypertriglyceridaemia (*p* = 0.442) and hypercholesterolaemia (*p* = 0.480) (Table 2). Three hypertriglyceridaemic subjects had hyperhomocysteinaemia (7.7%) while five hypercholesterolaemic subjects had hyperhomocysteinaemia (13.1%) (Table 3). The insignificant association was supported by the findings of Vayá *et al.*^15^ However, Nabipour *et al.* found significant associations between lower HDL cholesterol and high homocysteine levels.^20^

Homocysteine levels were estimated in 72 subjects with high systolic blood pressure and 84 subjects with high diastolic blood pressure, a total of 156 hypertensive subjects. Homocysteine was statistically significantly associated with both systolic (*p* = 0.002) and diastolic (*p* = 0.033) blood pressure (Table 2). Eleven hypersystolic subjects (15.3%) were hyperhomocysteinaemic while 16 hyperdiastolic subjects (19%) were hyperhomocysteinaemic (Table 3). These findings are supported by various researchers, who found hyperhomocysteinaemia to be significantly associated with hypertension.^10^,^20^-^22^

The association of homocysteine with hypertension may be due to the fact that homocysteine induces arteriolar constriction, renal dysfunction and increased sodium absorption, with increased arteriolar stiffness.^18^ It increases oxidative stress, which causes oxidative injury to the vascular endothelium, diminishing vasodilation by nitric oxide. It also stimulates the proliferation of vascular smooth muscle cells and alters the elastic properties of the vascular wall, leading to an increase in hypertension.^18^

On the basis of our findings, the large body of supporting evidence and the mechanisms of association, homocysteine levels can be used to track blood pressure. Hyperhomocysteinaemia reflects a causal effect rather than being concomitant to elevated blood pressure.

In our present study we evaluated 95 obese subjects for homocysteine association with obesity. We found a borderline association (*p* = 0.080) (Table 2). Ten obese subjects were hyperhomocysteinaemic (Table 3). The association was partly supported by other researchers, who found increased prevalence of hyperhomocysteinaemia in obese subjects.^10^,^15^,^36^ Depending on age and the pattern of obesity, homocysteine may be significantly associated with obesity. This viewpoint is supported by the findings of Vayá *et al*.^15^ and El-Sammak *et al*.^37^

## Conclusion

We found no statistically significant relationship between baseline plasma homocysteine levels and hyperglycaemia, dyslipidaemia and obesity. There was, however, a significant relationship between homocysteine levels and hypertension. According to our cross-sectional study, high baseline plasma homocysteine level is a major risk factor for hypertension and can be used in blood pressure tracking in a large, community-based sample. The study supported the hypothesis that plasma homocysteine is casually related to elevated blood pressure.

Additional prospective investigations are recommended to confirm these findings. A study evaluating the association between plasma homocysteine levels and hyperglycaemia after a few days of treatment withdrawal would probably yield better and more reliable results. Unfortunately, withdrawing treatment from diabetic subjects may be risky, especially in those with high glucose levels. We plan in future to compare homomocysteine levels between participants with and without the metabolic syndrome.

## References

[1] Shaikh MK, Devrajani BR, Shaikh A, Shah SZA, Shaikh S, Singh D (2012). Plasma homocysteine level in patients with diabetes mellitus. World App Sc J.

[2] Akalin A, Alatas O, Colak O (2008). Relation of plasma homocysteine levels to atherosclerotic vascular disease and inflammation markers in type 2 diabetic patients.. Eur J Endocrinol.

[3] Babic N, Dervisevic A, Huskic J, Music M (2011). Coagulation factor VIII activity in diabetes.. Med Glas Ljek Kamore Zenicko-doboj Kantona.

[4] Creager MA, Luscher TF, Consentino F, Beckman JA (2003). Diabetes and vascular disease: Pathophysiology, clinical consequences and medical therapy.. J Am Heart Assoc.

[5] Ford ES, Ajani UA, Croft JB, Critchley JA, Labarthe DR (2007). Explaining the disease in US death from coronary disease.. New Engl J Med.

[6] Lowe GDO (2004). Venous and arterial thrombosis: Epidemiology and risk factors at various ages.. Maturitas.

[7] Ntaios G, Savopoulos C, Grekas D, Hatzitolios A (2009). Homocysteine metabolism and causes of hyperhomocysteinaemia.. Arch Cardio Dis.

[8] Robertson J, Iemolo F, Stabler SP, Allen RH, Spence JD (2005). Vitamin B12, homocysteine and carotid plaque in the era of folic acid fortification of enriched cereal grain products.. Can Med Assoc J.

[9] Guilliams TG (2004). Homocysteine – a risk factor for vascular diseases.. J Am Neutraceut Assoc.

[10] Karatela RA, Sainani GS (2009). Plasma homocysteine in obese, overweight and hypertensive subjects in Mumbai.. Indian Heart J.

[11] Hajer GR, van der Graaf Y, Olijhoek JK, Verhaar MC, Visseren FJ (2007). Levels of homocysteine are increased in metabolic syndrome patients but are not associated with an increased cardiovascular risk, in contrast to patients without the metabolic syndrome.. Heart.

[12] Mishra PK, Tyagi N, Sen U, Joshua IG, Tyagi SC (2010). Synergism in hyperhomocysteinemia and diabetes: RHle of PPAR gamma and tempol.. Cardiovasc Diabetol.

[13] Schalinske KL (2003). Interrelationship between diabetes and homocysteine metabolism: Hormonal regulation of cystathionine beta-synthase.. Nutr Rev.

[14] Elias AN, Eng S (2005). Homocysteine concentrations in patients with diabetes mellitus – relationship to microvascular and macrovascular disease.. Diabetes Obesity Metab.

[15] Vayá A, Carmona P, Badia N, Pérez R, Hernandez MA, Corella D (2011). Homocysteine levels and the metabolic syndrome in a Mediterranean population.. Clin Hemorheol Microcirc.

[16] Wedro BC 2009 Blood pressure guidelines.. http://www.medicinenet.com/script/main/art.asp?articlekey=83068..

[17] Lim K, Steinberg G Preeclampsia. 2010.. http://emedicine.medscape.com/article/1476919-overview..

[18] Sen U, Tyagi SC (2010). Homocysteine and hypertension in diabetes: Does PPARγ have a regulatory role?. PPAR Res.

[19] Atif A, Rizvi MA, Tauheed S, Aamir I, Majeed F, Siddiqui K (2008). Serum homocysteine concentrations in patients with hypertension.. Pak J Physiol.

[20] Nabipour I, Ebrahim A, Jafari SM, Vahdat K, Assadi M, Movahed A (2009). The metabolic syndrome is not associated with homocysteinemia: The Persian Gulf healthy heart study.. J Endocrinol Invest.

[21] Sundström J, Sullivan L, D’Agostino RB, Jacques PF, Selhub J, Rosenberg IH (2003). Plasma homocysteine, hypertension incidence, and blood pressure tracking. The Framingham heart study. Hypertension.

[22] Stehouwer CD, van Guldener C (2003). Does homocysteine cause hypertension?. Clin Chem Lab Med.

[23] Brunzell JD, Davidson M, Furberg CD, Goldberg RB, Howard BV, Stein JH (2008). Lipoprotein management in patients with cardio-metabolic risk.. J Am Coll of Cardiol.

[24] Mahan LK, Escort-Stump S (2007). Food, Nutrition and Diet Therapy.

[25] Mahan LK, Escort-Stump S (2007). Food, Nutrition and Diet Therapy.

[26] Nelson DL, Cox MM (2008). Digestion, Mobilization, and Transport of Fats. 5th edn.

[27] Barter P, Gotto AM, Larosa JC, Maroni J, Szarek M (2007). HDL cholesterol, very low levels of LDL cholesterol and cardiovascular events.. N Engl J Med.

[28] Cziraky MJ, Watson KE, Talbert RL (2008). Targeting low HDL-cholesterol to decrease residual cardiovascular risk in managed care setting.. J Manag Care Pharm.

[29] Fahy E, Subramaniam S, Brown HA (2005). A comprehensive classification system for lipids.. J Lipid Res.

[30] Obeid R, Herrmann W (2009). Homocysteine and cholesterol: Guilt by association?. Stroke.

[31] Rippey FF (2003). Thrombosis and Embolism. General Pathology.

[32] Malhotra R, Hoyo C, Ostbye T, Hughes G, Schwartz D, Tsolekile L (2008). Deteminants of obesity in an urban township of South Africa.. J Clin Nutr.

[33] Darvall KA, Sam RC, Silverman SH, Bradbury AW, Adam DJ (2007). Obesity and thrombosis.. Eur J Vasc Endovasc Surg.

[34] Bodary PF, Randal J, Westrick BS, Wickenheiser KJ, Shen Y, Daniel T (2002). Effect of leptin on arterial thrombosis following vascular injury in mice.. J Am Med Ass.

[35] Goedecke JH, Jennings CL, Lambert EV (2006). Obesity in South Africa. Chronic diseases of lifestyle in South Africa since 1995–2005..

[36] Sanlier N, Yabanci N (2007). 2007. Relationship between body mass index, lipids and homocysteine levels in university students.. J Pak Med Assoc.

[37] EL Sammak M, Kandil M, EL-Hifni S, Hosni R, Ragab M (2004). Elevated plasma homocysteine is positively associated with age independent of C677T mutation of the methylenetetrahydrofolate reductase gene in selected Egyptian subjects.. Int J Med Sci.

